# Measurement of iron status in chronic kidney disease

**DOI:** 10.1007/s00467-018-3955-x

**Published:** 2018-04-17

**Authors:** Wesley Hayes

**Affiliations:** 1 Great Ormond Street Hospital, London, UK; 2 University College London Institute of Child Health, London, UK

**Keywords:** Iron, Anemia, Erythrocyte indices, Reticulocytes, Hepcidins, Chronic kidney disease

## Abstract

Anemia is a common complication of chronic kidney disease (CKD) in children, and dysregulation of iron homeostasis plays a central role in its pathogenesis. Optimizing iron status is a prerequisite for effective treatment of anemia. Insufficient iron can lead to inappropriate escalation of the erythropoiesis-stimulating agent (ESA) dose, which is associated with adverse outcomes. Excess iron supplementation also has negative sequelae including free radical tissue damage and increased risk of systemic infection. Notwithstanding the importance of optimizing bioavailable iron for erythropoiesis for children with advanced CKD, achieving this remains challenging for pediatric nephrologists due to the historical lack of practical and robust measures of iron status. In recent years, novel techniques have come to the fore to facilitate accurate and practical assessment of iron balance. These measures are the focus of this review, with emphasis on their relevance to the pediatric CKD population.

## Introduction

Anemia is a common complication of advanced chronic kidney disease (CKD), with prevalence exceeding 87% in children with CKD stages 4 and 5 [1, 2]. The pathogenesis and management of anemia in CKD were recently reviewed elsewhere [3]. Deficiency and dysregulation of iron play a central role [1, 3, 4].

Optimizing iron status is a prerequisite for effective treatment of anemia in children with CKD. If sufficient iron is not available for erythropoiesis, this can lead to inappropriate escalation of erythropoiesis-stimulating agent (ESA) therapy, which is associated with adverse outcomes in both adults and children [5–8]. Conversely, iron overload also has negative sequelae including free radical tissue damage, increased risk of systemic infection, and more hospitalizations [9–11]. Achieving the optimal balance of bioavailable iron for erythropoiesis in children with advanced CKD is therefore paramount in anemia management; however, it remains challenging for pediatric nephrologists. A key reason for this is the historical lack of practical yet robust measures of iron status. In recent years, novel techniques have come to the fore that can help to facilitate accurate and practical assessment of iron balance. These measures are the focus of this review.

## Iron homeostasis in health

A working knowledge of iron homeostasis in healthy children is helpful background for considering measures of iron status and their application to anemia management in CKD. The key processes responsible for regulation of iron absorption and release are reviewed in detail elsewhere [12, 13] and briefly summarized here.

**Fig. 1:**
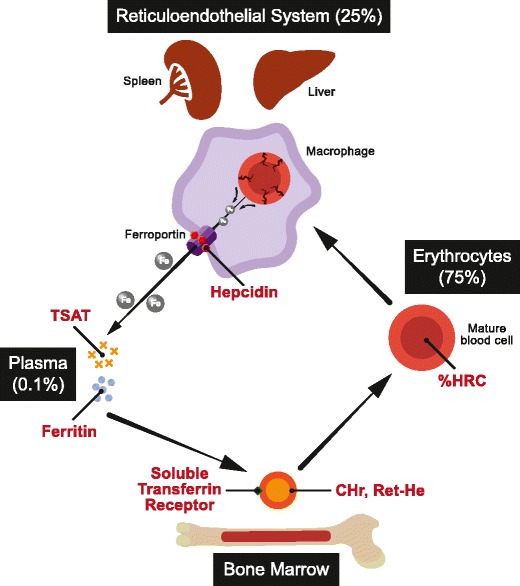
Iron recycling via the reticuloendothelial system. Approximate proportion of body iron stores in each system are indicated in parentheses. Iron measures are depicted in red text. *TSAT* transferrin saturation, *CHr* reticulocyte hemoglobin content, *Ret-He* reticulocyte hemoglobin equivalent, *%HRC* proportion of hypochromic red cells

Approximately 75% of the body’s iron circulates in erythrocytes, with a smaller proportion stored in hepatocytes for release when required. Less than 0.1% total body iron circulates in plasma bound to transferrin, its main transport protein. In health, daily iron intake and losses comprise just 0.05% total body iron and are tightly balanced via feedback control. Iron in erythrocytes is efficiently recycled via the reticuloendothelial system; macrophages phagocytose senescent erythrocytes and release controlled quantities of iron into the circulation, which is transported to the bone marrow via transferrin for incorporation into red cell precursors (Fig. 1).

Circulating iron levels are tightly regulated. Both import of iron via duodenal enterocytes to plasma and the release of recycled iron from macrophages and stored iron from hepatocytes to plasma are mediated by the cellular transport protein ferroportin [14]. Ferroportin expression is controlled by hepcidin, the hormone chiefly responsible for extracellular iron regulation. Hepcidin negatively regulates plasma iron levels by binding to ferroportin and inducing its internalization [15].

Although iron stores and bioavailable iron in plasma are tightly regulated in health, iron homeostasis is disturbed in CKD. The underlying reasons and their relevance to iron measurements will now be outlined.

## Iron dysregulation in CKD

Iron deficiency in children with CKD can be thought of as either absolute, functional, or a combination of both. Absolute iron deficiency refers to insufficient body iron stores. Functional deficiency refers to a situation in which the bioavailability of iron for incorporation into reticulocytes is compromised, despite normal or excessive total body iron stores.

Absolute iron deficiency in CKD can result from insufficient dietary iron intake or duodenal iron absorption, and/or from excessive iron loss, for example through extracorporeal hemodialysis circuits or gastrointestinal sloughing. Functional iron deficiency results from impaired extracellular iron homeostasis. In recent years, hepcidin dysregulation has been found to play a key role in the functional iron deficiency in CKD. Hepcidin is a 2.7-kDa protein which is filtered by the kidneys [16]. Hepcidin clearance is therefore reduced in CKD disrupting its regulation [17]. A further reason for inappropriate elevation in hepcidin is chronic inflammation, a well-recognized complication of CKD [18]. The net effect of reduced renal clearance, and inflammatory upregulation, is chronic inappropriate elevation in hepcidin concentration resulting in suppression of both duodenal absorption of iron and its release from macrophages and hepatocytes.

The combined effect of reduced iron absorption and release results in a net reduction in iron available for incorporation into emerging reticulocytes in the bone marrow, resulting in iron-restricted erythropoiesis. This results in a suboptimal response to ESA, which can lead to inappropriate escalation of ESA doses if suitable assessment of bioavailable iron is not undertaken to inform iron supplementation.

## Aims of iron measurement

Optimizing iron balance in children with CKD involves ensuring availability of sufficient iron for red cell precursors to use as raw material for erythropoiesis, while avoiding excessive iron supplementation and its sequelae. Measures of iron status should therefore indicate the amount of bioavailable iron to red blood cell precursors for incorporation into hemoglobin; they should also indicate iron excess. Minimizing blood sampling is also an important consideration in children, since frequent sampling can exacerbate iron depletion, particularly in infants for whom blood samples constitute a greater proportion of iron stores. Cost is another important factor in healthcare systems that are increasingly budget constrained. Acceptable levels of variability, both biological and analytical, are also key requirements for a robust measure of iron status.

A key challenge in developing suitable measures of iron in patients with CKD is the lack of an established gold standard comparator. Previously, iron staining of bone marrow biopsy specimens was considered the gold standard for assessment of iron stores. This is unsuitable for children with CKD for a number of reasons. Firstly, bone marrow iron staining is not a robust test; it has significant variability and is dependent on the operator and process used. Secondly, sampling is a painful procedure that requires a general anesthetic in the majority of children. Thirdly, bone marrow biopsy is impractical to repeat on a regular basis as would be required for iron monitoring in children on dialysis. Due to the impracticality of assessing bone marrow iron stores, studies on adult patients have used clinical parameters as gold standard markers of iron repletion, most commonly the response in hemoglobin to ESA. This is currently the most clinically relevant and practical standard for evaluating iron measures in children with CKD.

In summary, iron measures for children with CKD should reliably quantify bioavailable iron for erythropoiesis as reflected in clinical erythropoietic response to ESA. Traditional and novel iron measures will now be discussed with consideration of these requirements.

## Limitations of traditional iron measures

Traditionally, a combination of transferrin saturation (TSAT) and serum ferritin was used to assess iron status in adults and children with CKD, and these measures are still recommended in The National Kidney Foundation’s Kidney Disease Outcomes Quality Initiative (KDOQI) and Kidney Disease: Improving Global Outcomes (KDIGO) anemia guidelines [19, 20]. There are however significant limitations to both measures that compromise their clinical utility.

### Biological and analytical variability

Biological variability refers to differences in the measured parameter between patients, and in the same patient measured at different times, resulting from physiological variation in a short period of time. Analytical variability refers to differences in test results performed on the same sample resulting from variability in the testing process.

Serum ferritin and TSAT have been shown to exhibit a high degree of both analytical and biological variability in adult patients with end-stage kidney disease (ESKD) [21, 22]. This is one of the key reasons underlying the recommendation by the National Institute for Health and Care Excellence not to use these parameters alone to assess iron status in CKD [23].

### Inflammation confounds measurements

Both serum ferritin and TSAT measurements are confounded by inflammation. Chronic inflammation is a feature of CKD in both adults and children and is partly related to suboptimal nutrition and dialysis [24–26]. Infections are a further source of inflammation and are a particular issue in children with central venous catheters or peritoneal dialysis access [27, 28]. Inflammation in children with CKD and ESKD compromises the reliability of serum ferritin and TSAT to reliably reflect patients’ iron status.

Ferritin is an intracellular iron storage protein. Its concentration in serum is influenced by several factors, one of which is intracellular iron stores. Serum ferritin is also a marker of acute inflammation and has a key role in the diagnosis of systemic inflammatory processes such as macrophage activation syndrome [29]. Serum ferritin is also affected by patients’ nutritional status as observed in adult hemodialysis patients [30]. Given significant confounding influences, the utility of ferritin as a marker of iron status in patients with kidney disease has been questioned for over a decade [31].

Transferrin saturation is not itself measured, but rather derived from measurements of serum iron and total iron binding capacity (TIBC). TIBC is a negative acute-phase reactant, that is, its plasma concentration is suppressed by inflammation. Low TIBC levels are associated with increased mortality in adult patients on dialysis [32, 33]. In the context of systemic inflammation, reductions in TIBC lead to higher levels of TSAT independent of patients’ iron status. Inflammation is therefore implicated in the poor reliability of TSAT as a measure of iron status in CKD.

### Comorbidity confounds measurements

Malnutrition is fortunately uncommon in pediatric nephrology units with adequate dietetic support; however, it is clearly associated with elevated serum ferritin levels in adults with CKD independent of iron status [30].

Liver disease also confounds serum ferritin measurements. The release of ferritin from intracellular stores into the circulation is incompletely understood; however, its clearance from the circulation depends largely on the reticuloendothelial system, of which the liver is a key part. In the context of liver dysfunction, e.g., due to hepatitis or fatty liver disease, reticuloendothelial clearance of circulating ferritin is impaired, resulting in higher serum ferritin levels. This is independent of iron status and therefore confounds the assessment of iron status in patients with liver disease.

Malignancy affects serum ferritin levels to such an extent that they are used as a tumor marker for both neuroblastoma and Hodgkin’s disease in children [34–36]. For this reason, it cannot be used to assess iron status in patients with malignancy.

Given the numerous confounding influences on both serum ferritin and TSAT in children with CKD and ESKD, the reliability of these traditional measures of iron status is poor. Alternative measures aimed at reliably reflecting bioavailable iron for erythropoiesis will now be discussed.

## Alternative measures of iron status

A number of measures to estimate bioavailable iron for incorporation into emerging erythrocytes have been developed in recent years. They are illustrated in Fig. 1 and outlined here.

### Reticulocyte hemoglobin content and reticulocyte hemoglobin equivalent

The application of automated blood analyzers to measure the iron content of circulating red cells to improve assessment of iron status was suggested by Macdougall over 25 years ago [37]. Subsequently, parameters which reflect availability of iron to developing red cells in the short term have been developed. Reticulocyte hemoglobin content is a measure introduced in Siemens blood count analyzers that quantify hemoglobin mass in reticulocytes. Given that hemoglobin production is directly dependent on bioavailable iron, and that reticulocytes mature into erythrocytes within days, the hemoglobin content of emerging erythrocytes reflects short-term changes in iron availability for erythropoiesis.

A related measure to assess iron bioavailability for emerging erythrocytes is the reticulocyte hemoglobin equivalent, which is available in Sysmex blood analyzers. Automated cell counts are undertaken using fluorescent markers to identify cellular RNA. Red blood cells, reticulocytes, and platelets are identified, and reticulocyte hemoglobin equivalent calculated from a combination of forward scatter and fluorescence.

Reticulocyte hemoglobin content (CHr) and reticulocyte hemoglobin equivalent (Ret-He) are both physiologically appropriate markers of iron status for children with CKD since they measure a short half-life product whose synthesis is highly dependent on iron availability. Additional advantages include less blood sampling (analysis is performed on the same EDTA sample used for full blood count analysis) and reduced cost compared to traditional measures (74% cost saving in our center).

Several studies in adult patients, and a small number of pediatric studies, support the use of CHr or Ret-He in the assessment of iron status in CKD. Their biological and analytical variability is superior to traditional iron measures [21]. In a study on 78 adult patients undergoing bone marrow examination, CHr and traditional measures of iron were compared to bone marrow iron staining as a gold standard measure of iron deficiency; receiver operating curves showed improved diagnostic performance of CHr [38]. CHr and Ret-He were superior to conventional iron measures in detecting iron-deficient erythropoiesis defined by erthropoietic response to intravenous iron therapy in studies on adult hemodialysis patients [39, 40]. Randomized controlled trials of CHr compared to TSAT showed decreased intravenous iron use with no significant difference in ESA doses when CHr was used to assess iron status [41, 42]. Ret-He was found to be both sensitive and specific in distinguishing iron deficiency anemia from other causes in adults with various pathologies [43].

A number of small pediatric studies have evaluated the utility of CHr as a maker of iron status. CHr was found to be a suitable marker of latent iron deficiency in preterm and very low birthweight infants; however, a key limitation was the use of traditional extracellular markers of iron as the gold standard comparator [44]. Ret-He was found to be a useful predictor of response to oral iron supplementation in 34 children with normal kidney function [45]. A study evaluating CHr in children with inflammatory bowel disease was inconclusive as traditional markers were used as a comparator, which are confounded by inflammation [46]. In a retrospective analysis of children on chronic dialysis, CHr was compared to traditional markers of iron status with the conclusion that prospective studies for further evaluation are warranted [47].

Pediatric reference ranges for CHr and Ret-He have been established [48, 49]. There is now sufficient evidence for the clinical utility of CHr and Ret-He that their use is recommended for the assessment of iron status in patients with CKD in European best practice guidelines [50] and UK NICE guidelines on the anemia in CKD [23].

### Proportion of hypochromic red cells

The proportion of hypochromic red cells, defined as cells with hemoglobin concentration < 280 g/L, was proposed as a marker of functional iron deficiency over 25 years ago [37]. Reduction in mean cell hemoglobin results after several weeks of insufficient hemoglobin production as a result of inadequate iron supply.

In adult patients on hemodialysis, proportion of hypochromic red cells (%HRC) performed better than traditional measures in a study performed before widespread availability of reticulocyte markers [51]. %HRC is now a well-established measure of functional iron deficiency, although it is not suitable for assessing short-term changes in iron status [52]. Given the published evidence for the clinical utility of %HRC, this measure is recommended in the UK NICE guideline for anemia management in people with CKD [23].

### Red blood cell size factor

The red blood cell size factor is the geometric mean of the mean cell volumes of mature red blood cells and reticulocytes. It was evaluated in healthy adults and those with anemia from various causes and found to correlate well with CHr with potential clinical utility in the diagnosis of iron-restricted erythropoiesis [53]. Published data in patients with CKD are lacking; it is therefore not currently recommended for clinical assessment of iron status in patients with kidney disease.

### Soluble transferrin receptor

The transferrin receptor is expressed on red cell precursors. It facilitates the incorporation of circulating transferrin-bound iron into erythrocytes. Elevated soluble transferrin receptor levels in plasma are associated with iron deficiency [54]. Limitations of soluble transferrin receptor as a marker of iron status include confounding by the use of ESAs, cost, and lack of widespread availability [52].

Studies of the use of soluble transferrin receptor to detect iron deficiency in adult patients with CKD have shown variable results, but in general, it was found to be an inferior marker to cellular measures such as %HRC or CHr [51, 55]. Combining soluble transferrin receptor with ferritin concentration in the soluble transferrin receptor index improves its performance; however, it remains inferior to cellular markers [56].

### Hepcidin

As outlined above, hepcidin is a protein synthesized by the liver that regulates both absorption of iron from the intestine and release of intracellular iron into plasma from reticuloendothelial cells following recycling of erythrocytes [12]. Expression of hepcidin is upregulated in iron overload and inflammation and downregulated in iron deficiency.

Early studies have evaluated the utility of hepcidin levels as a measure of functional iron deficiency in CKD. In a study of 78 adults with CKD stages 3 to 4, lower hepcidin levels were predictive of erythropoietic response to intravenous iron supplementation, albeit with sensitivity 84% and specificity of only 48% [57]. Clinical data and availability of hepcidin measurement are however limited, and it remains a research tool to date.

### Zinc protoporphyrin

Zinc protoporphyrin is a by-product of heme synthesis, and its concentration in red cells increases when iron supply to emerging reticulocytes is reduced [58]. It has therefore been evaluated as a potential measure of bioavailable iron for erythropoiesis. Key limitations of zinc protoporphyrin include confounding of results by hyperbilirubinemia, reduced kidney function, and severe anemia [59]. These can be partly mitigated by washing red cells prior to testing, although this is expensive and not entirely practical for a routine clinical test.

Studies in adult patients evaluating the clinical utility of zinc protoporphyrin as a measure of iron status have conflicting results. While it has been reported to be predictive of iron deficiency in hemodialysis patients [60, 61], further studies found that it did not change after intravenous iron treatment and did not correlate with bone marrow iron stores [62, 63]. It is therefore not recommended in the routine clinical assessment of iron status in patients with CKD.

## Clinical guidelines

National and international guidelines vary in their recommendations for iron measurement in anemia of CKD as summarized in Table 1. Prior to 2004, serum ferritin and TSAT were recommended in all guidelines and used globally to assess iron status. Since then, European, NICE, and British Committee for Standards in Haematology guidelines endorse alternative measures of %HRC, CHr, and Ret-He. KDOQI and KDIGO guidelines still recommend ferritin and TSAT, which predominate in US clinical practice.

**Table 1:** International guidelines on iron assessment in chronic kidney disease (CKD)

Guideline	Recommended iron measures	References
KDOQI anemia guideline 2006, and 2007 amendment with revised hemoglobin target	Serum ferritin and TSAT in adults with non-dialysis CKD and on peritoneal dialysis; for adults on hemodialysis, either CHr or TSAT in combination with serum ferritin	[5, 19]
KDIGO guideline 2012	Ferritin and TSAT	[20]
European guidelines 2004	%HRC, TSAT, or CHr	[50]
NICE anemia guideline 2015 update	%HRC if processing of the blood sample is available within 6 h, or CHr or Ret-He if %HRC is not available	[23]
The British Committee for Standards in Haematology guideline for the laboratory diagnosis of functional iron deficiency 2013	%HRC is the best established variable for identification of functional iron deficiency; CHr and Ret-He have predictive value for the likelihood of response to intravenous iron therapy in patients on hemodialysis; low serum ferritin has a role in the diagnosis of functional iron deficiency; TSAT alone is not recommended as a predictor of responsiveness to intravenous iron therapy	[52]

*KDOQI* The National Kidney Foundation’s Kidney Disease Outcomes Quality Initiative, *TSAT* transferrin saturation, *CHr* reticulocyte hemoglobin content, *Ret-He* reticulocyte hemoglobin equivalent, *KDIGO* Kidney Disease: Improving Global Outcomes, *%HRC* proportion of hypochromic red cells

The geographical variation in guidelines and clinical adoption of cellular measures of iron status may be due to a key limitation of these measures, namely confounding with sample storage. Measuring %HRC is unreliable if blood samples are not processed within 6 h, because cells swell during storage. Use of national laboratories by most of the large dialysis chains in the USA incurs a processing delay of 18 to 24 h due to sample shipping [64]. This delay confounds CHr, Ret-He, and %HRC measures and is a key factor hindering their adoption. Implementation of these measures is more straightforward in Europe where processing delays are minimized by using local laboratories.

## Special situations

There are certain situations in which iron measures need to be interpreted with caution:

Red cell disorders such as thalassemia and glucose-6-phosphate dehydrogenase (G6PD) deficiency confound cellular measures such as CHr, Ret-He, %HRC, and red blood cell size factor. In these conditions, atypical features of both hemoglobin and red cells render cellular indices non-interpretable. Zinc protoporphyrin is also elevated in thalassemia traits and is therefore not useful for iron assessment in patients with hemoglobinopathies.

Iron measures should be also interpreted with caution in the postnatal period, as the effect of changing hemoglobin composition from HbF to HbA on cellular measures of iron has not been studied. We are currently gathering data with the aim of establishing a reference range for Ret-He in infants under 6 months of age.

Caution is needed if patients’ iron status is assessed following transfusion of packed red blood cells, which deliver a bolus of 220–250 mg iron per adult unit transfused [65]. Measurement of %HRC is confounded by red cell transfusion because infused exogenous normochromic red cells artificially reduce the proportion of circulating hypochromic cells. CHr and Ret-He are less affected by transfusion of mature erythrocytes; however, assessment of iron status is rarely helpful immediately following red cell transfusion because of the associated iron bolus.

## Summary

Careful assessment of iron availability for erythropoiesis is important for children with CKD in order to optimize anemia management and avoid sequelae of iron deficiency (such as morbidity from excessive ESA doses), and excessive iron (organ toxicity and increased infection). Traditional measures of iron, serum ferritin and TSAT, are not fit for this purpose due to unacceptable analytical and biological variability, and confounding by inflammation, nutritional status, and other co-morbidities. Novel measures such as %HRC, CHr, and Ret-He offer superior assessment with additional advantages of reduced blood sampling and cost saving and are now recommended in European and UK NICE guidelines for assessment of anemia in children with CKD.

Notwithstanding these advances, no single parameter offers comprehensive assessment of body iron stores and bioavailable iron for erythropoiesis. Caution is required when interpreting iron measures in all babies in the postnatal period, and in children with erythrocyte disorders such as thalassemia. The absorption, storage, recycling, and transport of iron involve multiple interrelated control mechanisms. Looking ahead, multi-parameter algorithms incorporating patients’ age, clinical features, and a panel of iron-related measures will be desirable to further enhance the assessment of iron status in children with CKD.

## Multiple choice questions (answers are provided following the reference list)

1. Key determinants of serum ferritin include:
  - Intracellular iron stores
  - Nutritional status
  - Inflammatory status
  - Macrophage activity
  - All of the above
2. UK NICE guidance for assessment and management of anemia in people with CKD recommends measurement of iron status with:
  - CHr and TSAT
  - Ret-He and zinc protoporphyrin
  - Ferritin, TSAT, and free serum iron
  - %HRC, or CHr/Ret-He if analysis of %HRC is not available within 6 h
  - None of the above
3. Hepcidin mediates:
  - Endocytosis of ferroportin
  - Increased absorption of iron from the duodenum
  - Reduced release of iron from macrophages to plasma
  - Increased release of iron from hepatocyte stores
  - a and c
4. Daily iron losses comprise the following proportion of body stores in health:
  - < 0.1%
  - 0.1–1%
  - 1–10%
  - > 10%
5. Complications of excess iron supplementation include:
  - Increased risk of infection
  - Mortality in adults with ESKD
  - Tissue toxicity
  - Gastrointestinal side effects
  - All of the above
